# Maltol prevents the progression of osteoarthritis by targeting PI3K/Akt/NF‐κB pathway: In vitro and in vivo studies

**DOI:** 10.1111/jcmm.16104

**Published:** 2020-11-19

**Authors:** Hongwei Lu, Changchang Fu, Suyan Kong, Xudong Wang, Lin Sun, Zeng Lin, Peng Luo, Haidong Jin

**Affiliations:** ^1^ Department of Orthopaedics The Second Affiliated Hospital and Yuying Children's Hospital of Wenzhou Medical University Wenzhou China; ^2^ The Second School of Medicine Wenzhou Medical University Wenzhou China; ^3^ The Key Orthopaedic Laboratory of Zhejiang Province Bone Research Institute Wenzhou China; ^4^ Department of Neonatology The Second Affiliated Hospital and Yuying Children's Hospital of Wenzhou Medical University Wenzhou China

**Keywords:** chondrocytes, inflammation, maltol, NF‐κB, osteoarthritis

## Abstract

Osteoarthritis (OA), a prevalent degenerative arthritis disease, principle characterized by the destruction of cartilage and associated with the inflammatory response. Maltol, a product formed during the processing of red ginseng (Panax ginseng, CA Meyer), has been reported to have the potential effect of anti‐inflammatory. However, its specific mechanisms are not demonstrated. We investigated the protective effect of maltol in the progression of OA both in vitro and in vivo experiments. Human chondrocytes were pre‐treated with maltol (0, 20, 40, 60 μM, 24 hours) and incubated with IL‐1β (10 ng/mL, 24 hours) in vitro. Expression of PGE2, TNF‐α and NO was measured by the ELISA and Griess reaction. The expression of iNOs, COX‐2, aggrecan, ADAMTS‐5, MMP‐13, IκB‐α, p65, P‐AKT, AKT, PI3K and P‐PI3K was analysed by Western blotting. The expression of collagen II and p65‐active protein was detected by immunofluorescence. Moreover, the serious level of OA was evaluated by histological analysis in vivo. We identified that maltol could suppress the IL‐1β‐stimulated generation of PGE2 and NO. Besides, maltol not only suppressed the production of COX‐2, iNOs, TNF‐α, IL‐6, ADAMTS‐5, MMP‐13, but also attenuated the degradation of collagen II and aggrecan. Furthermore, maltol remarkably suppressed the phosphorylation of PI3K/AKT and NF‐κB induced by IL‐1β in human OA chondrocytes. Moreover, maltol could block the cartilage destroy in OA mice in vivo. To date, all data indicate maltol is a potential therapeutic agent by inhibiting inflammatory response via the regulation of NF‐κB signalling for OA.

## 1 INTRODUCTION

Osteoarthritis (OA), an irreversible degenerative arthritis disease, caused by severe joint instability, with main pathological features of progressive of subchondral bone sclerosis, articular cartilage, osteophyte formation and synovial inflammation.^1^ It is regarded as the most commonly prevalent form of chronic arthritis and affects daily activities, particularly among old people.^2^, ^3^ Many studies found a series of factors influence the development of osteoarthritis including ageing, trauma, obesity, inflammation, joint malformation and osteoporosis.^4^ However, the pathogenesis of OA is still murky. It is confirmed that inflammation and oxidative stress are vital risk factors in the progression of OA.^5^ Many factors of OA factors also can imbalance the oxidant‐antioxidant levels and then stimulates chondrocytes to produce inflammatory cytokines.^6^, ^7^, ^8^ Inflammation cytokines including tumour necrosis factor‐α (TNF‐α) and interleukin‐6 (IL‐6) are involved in the pathogenesis of OA.^9^ IL‐1β boosts the degradation of extracellular matrix (ECM) in the way of inducing the release of pro‐inflammatory mediators, such as nitric oxide (NO), prostaglandin E2 (PGE2), matrix metalloproteinases (MMPs) and a disintegrin and metalloproteinase thrombospondin motifs (ADAMTS).^10^, ^11^ Therefore, anti‐inflammatory has been demonstrated as a valid therapeutic strategy for attenuating the development of OA.

The NF‐κB signalling pathway has been demonstrated to play a core role in catabolism and inflammatory response.^12^ When initiated by certain stimuli such as IL‐1β, the action of IκBα is caused by a succession of membrane‐proximal events.^13^ NF‐κB p65 translocates from the cytoplasm to the nucleus, which stimulates the production of inflammatory genes, including TNF‐α, iNOS, PGE2, NO, COX‐2, IL‐6, ADAMTS and MMPs.^14^ Many studies found that inhibition of NF‐κB could attenuate the development of OA by suppressing the activity of the PI3K/Akt signalling pathway, which is one of the most significant upstream factors of the NF‐κB signalling pathway.^15^, ^16^ They all related to the degradation of the extracellular matrix. Consequently, the PI3K/AKT/NF‐κB signalling pathway is a potential therapeutic option in the progression of OA.

Maltol (3‐hydroxy‐2‐methyl‐4‐pyrone), a safe and reliable flavour enhancer, is a product of the Maillard reaction of heated‐processed ginseng.^17^ It is also existed in the roasted Korean ginseng root.^18^ In these years, it is recognized that maltol prevents renal injuries by suppressing PI3K/AKT signalling pathway.^19^ What's more, maltol has the protective property for oxidative stress such as glaucoma and attenuates liver apoptosis inflammation by inhibiting the NF‐κB signalling pathway.^20^ Nevertheless, the protective effect of maltol in OA is still obscure. In our research, we demonstrated whether maltol exerted a protective role in OA progression and explored the potential mechanism both in vitro and vivo.

## 2 MATERIALS AND METHODS

### 2.1 Reagents

Maltol (purity ≥ 98%), collagenase II, Safranin‐O/Fast Green and dimethylsulphoxide (DMSO) were obtained from Solarbio. Primary antibodies of Aggrecan, iNOS, Collagen type II, and ADAMTS5 were obtained from Abcam. Primary antibodies directed against LaminB, p65 and IkBα were gained from Cell Signaling Technology. Primary antibodies against COX‐2 and GAPDH were procured from ProteinTech. Cell‐ foetal bovine serum (FBS) and Dulbecco's modified Eagle's medium (DMEM)/F12 were obtained from Gibco. Recombinant human IL‐1β was obtained from Novoprotein. Counting Kit‐8 (CCK‐8) was gained from Dojindo. The second antibodies of Goat Anti‐Rabbit IgG, Goat Anti‐Mouse IgG Alexa Fluor^®^594 and Alexa Fluor^®^488 labelled were obtained from Bioworld. Bovine serum albumin (BSA) was procured from Beyotime Biotechnology. TRIzol reagent was purchased from Invitrogen. The BCA protein assay kit was procured from Beyotime.

### 2.2 Primary human chondrocyte isolation and culture

Human articular cartilage tissue collection with OA was performed by the Medical Ethical Committee of the Second Affiliated Hospital (ethic cord: LCKY‐2018‐27), Wenzhou Medical University and followed the guidelines of the Declaration of Helsinki and Tokyo. Firstly, human cartilage tissues were procured from ten OA patients (age 54 ± 8 years, five men and five women) who had undergone total knee replacement surgery at the Second Affiliated Hospital of Wenzhou Medical University. Secondly, cartilage tissues were cut into 1 × 1 × 1 mm^3^ pieces and washed with PBS for three times. The pieces were in collagenase II (2 mg/mL) for 4 hours at 37°C. After centrifuging at 800 rpm for 6 minutes, the cells were cultured in DMEM/F12 medium with 10% FBS under an atmosphere containing 5% CO2 at 37°C. The cells were changed every other day. The human cells were passaged using 0.25% trypsin‐EDTA solution at 80 to 90% confluence.

### 2.3 CCK‐8 assay

The cytotoxicity of Maltol on human chondrocytes was assayed by the Cell Counting Kit‐8 obeying the protocols of the manufacturer. Firstly, human chondrocytes were cultured in 96‐well plates (5000 cells/well) for 24 hours and incubated with various concentrations of maltol (0, 10, 20, 40, 60, 80 and 100 μM) for 24 hours or 48 hours. Afterwards, the chondrocytes followed by rinsing in PBS thrice. Then, we added 10 μL CCK‐8 solution to each well and incubated 96‐well plates at 37°C for 3 hours. The absorbance of the wells was then detected at a wavelength of 450 nm using a microplate reader (Leica MicrosystemGermany).

### 2.4 Griess reaction and ELISAs

The cells (3 × 10^5^ cells/mL) were cultured in 6‐well plates, pre‐treated with maltol (0, 20, 40 or 60 μM). After 24 hours, they were added IL‐1β (10 ng/mL) and then incubated for 24 hours. The content of NO in each well was appraised by using the Griess reaction. The concentration of PGE_2_, tumour necrosis factor‐α (TNF‐α) and interleukin‐6 (IL‐6) in each well was detected by ELISA kits.

### 2.5 qRT‐PCR

After stimulated with IL‐1β (10 ng ml^−1^) and treated with various concentrations of maltol (0, 20, 40 and 60μM), the total RNA in the human chondrocytes was gained through using TRIzol Reagent (Invitrogen). Quantitative real‐time PCR (qPCR) was carried out using CFX96 real‐time PCR system (Bio‐Rad Laboratories). under the following conditions: 10 minutes 95°C, followed by 40 cycles of 15 seconds 95°C and 1 minutes 60°C. The reaction was carried out in a total of 10 µL, containing 5 µL of 2 × SYBR Master Mix, 0.25 µL of each primer and 4.5 µL of diluted cDNA. Cycle threshold (Ct) values were collected and normalized to the GAPDH levels. Relative mRNA levels of each target gene were calculated using the 2 − ΔΔCt method. Primers of iNOS, COX‐2, IL‐6 and TNF‐α were designed with the help of NCBI Primer‐Blast Tool, which was listed below: iNOS (F) 5′GACGAGACGGATAGGCAGAG‐3′, (R) 5′‐CACATGCAAGGAAGGGAACT‐3′; COX‐2 (F) 5′‐TCCTCACATCCCTGAGAACC‐3′, (R) 5′.

GTCGCACACTCTGTTGTGCT‐3′; IL‐6, (F) 5′‐CCGGAGAGGAGACTTCACAG‐3′, (R) 5′‐TCCACGATTTCCCAGAGAAC‐3′; TNF‐α(F) 5′‐ACGGCATGGATCTCAAAGAC‐3′, (R) 5′.

GTGGGTGAGGAGCACGTAGT‐ 3′.

### 2.6 Western blotting

The expression level of protein was measured by Western blotting. The proteins were separated by using RIPA lysis buffer (1 mM PMSF) and then sonicated on ice for 10 minutes and then centrifuged at  4000 g at 4°C for 15 minutes. Then, the protein concentration was evaluated via the BCA protein assay kit (Beyotime). The protein (40 mg) was separated by sodium dodecyl sulphate (SDS)‐polyacrylamide gel electrophoresis, followed by transferring to a polyvinylidene difluoride (PVDF) membrane (Millipore). After blocking with 5% non‐fat milk for 3 hours, the obtained membranes were incubated with the primary antibodies: IκBα (1:2000), p65 (1:2000), COX‐2 (1:2000), iNOS (1:2000), GAPDH (1:2000), Lamin B (1:2000), AKT (1:2000), p‐AKT (1:2000), PI3K (1:2000) and p‐PI3K (1:2000). The following step was that incubating the secondary antibodies at room temperature for 2.5 hours. The blots were visualized via the Imaging System (Bio‐Rad) followed by washing with TBST 3 times.

### 2.7 Analysis of immunofluorescence

The cells rinsed with PBS and treated with 4% paraformaldehyde fixation for 15 minutes; then, the wells were washed with PBS 3 times again. Then, we treated with 0.1% Triton X‐100 diluted in PBS for 15 minutes at indoor temperature. Next, human chondrocytes were blocked with 10% goat serum and incubation with primary antibodies against collagen II (1:300) and p65 (1:300) for the whole night at the temperature of 4°C. The next day, cells were exposed to Alexa Fluor^®^594 and Alexa Fluor^®^ 488‐labelled conjugated secondary antibodies (1:400) for 1.5 hours. Finally, the cells were exposed to DAPI (Beyotime) for 1 minutes. Ultimately, cell samples were detected on the Olympus fluorescence microscope. The fluorescence intensity was observed by using Image J software.

### 2.8 X‐ray imaging assay

The mice took on the X‐ray machine After 8 weeks of experiments. We performed X‐ray imaging on all of the mice to assess osteophyte formation, the joint space and superficial cartilage changes detected by a digital X‐ray machine (KUB Technologies Inc.) with the following settings: 50 Kv and 160 μA.

### 2.9 Mice OA models

Forty‐five ten‐week‐old C57BL/6 male wild‐type (WT) mice were obtained from the Animal Center of Chinese Academy of Sciences Shanghai, China. The protocols of animal use and care and the experimental procedures were accord with the Animal Care and Use Committee of Wenzhou Medical University (ethic code: wydw2019‐0808). The mice OA models were established by surgical destabilization of the medial meniscus (DMM).^21^ Briefly, mice were anaesthetized with intraperitoneal injection of 2% (w/v) pentobarbital (30 mg kg^−1^), followed by cutting the right knee joint capsule inside the medial Malleolus and the medial meniscus ligament with microsurgical scissors. An operation of arthrotomy without the transaction of medial meniscus ligament was as well carried out in the left knee joint of mice as a sham operation group. After the operation, the mice were randomly divided into three groups (n = 15): sham control group, an OA group (DMM) and an OA treated with the maltol group (DMM + maltol).

After DMM, the maltol treatment group received a dose of maltol for 100 mg·kg^−1^·day^−1^ (dissolved in saline, administered by oral gavage every day for 8 weeks).^44^ Simultaneously, mice in the DMM alone group received an equal amount of saline. All animals (15 mice/group) were killed 8 weeks after the surgery, and cartilage samples were collected for immunological and histological analysis.

### 2.10 Histopathologic analysis

Safranin‐O/Fast Green was used to evaluate the articular cartilage destruction. Then, we utilized a light microscope to assess the morphologic changes of mouse chondrocytes and surrounding tissues, and the Osteoarthritis Research Society International (OARSI) scoring system was used to assess the extent of cartilage destruction as described previously.

### 2.11 Immunohistochemical assay

The Knee joints were fixed in 4% paraformaldehyde, decalcified, embedded in paraffin, and cut into 7‐μm sections that were deparaffinized, rehydrated. Then, the sections were treated with 3% (v/v) hydrogen peroxide and 0.25% trypsin‐EDTA solution at 37°C for 30 minutes. Next, the histological sections were incubated 10% bovine serum albumin for 60 minutes at 37°C. Treated with the primary antibody against Collagen II and P65 for 24 hours at 4°C. On the second day, the sections were incubated with HRP‐conjugated secondary antibody for 1 hour at 4°C. Images were analysed by Image‐Pro Plus software, version 6.0 (Media Cybernetics). Five sections from each group were used for quantitative analysis.

### 2.12 Statistical analysis

The experiments were required to be at least performed five times. The data obtained are expressed as the mean ± standard deviation. Statistical analyses were performed using GraphPad Prism version 5.0 software (GraphPad software, San Diego, CA, USA). Inter‐group comparisons were performed using a one‐way ANOVA followed by the Tukey test. Probability values of *P* < .05 were considered statistically significant.

## 3 RESULT

### 3.1 Effect of maltol on human chondrocytes viability

**Figure 1:**
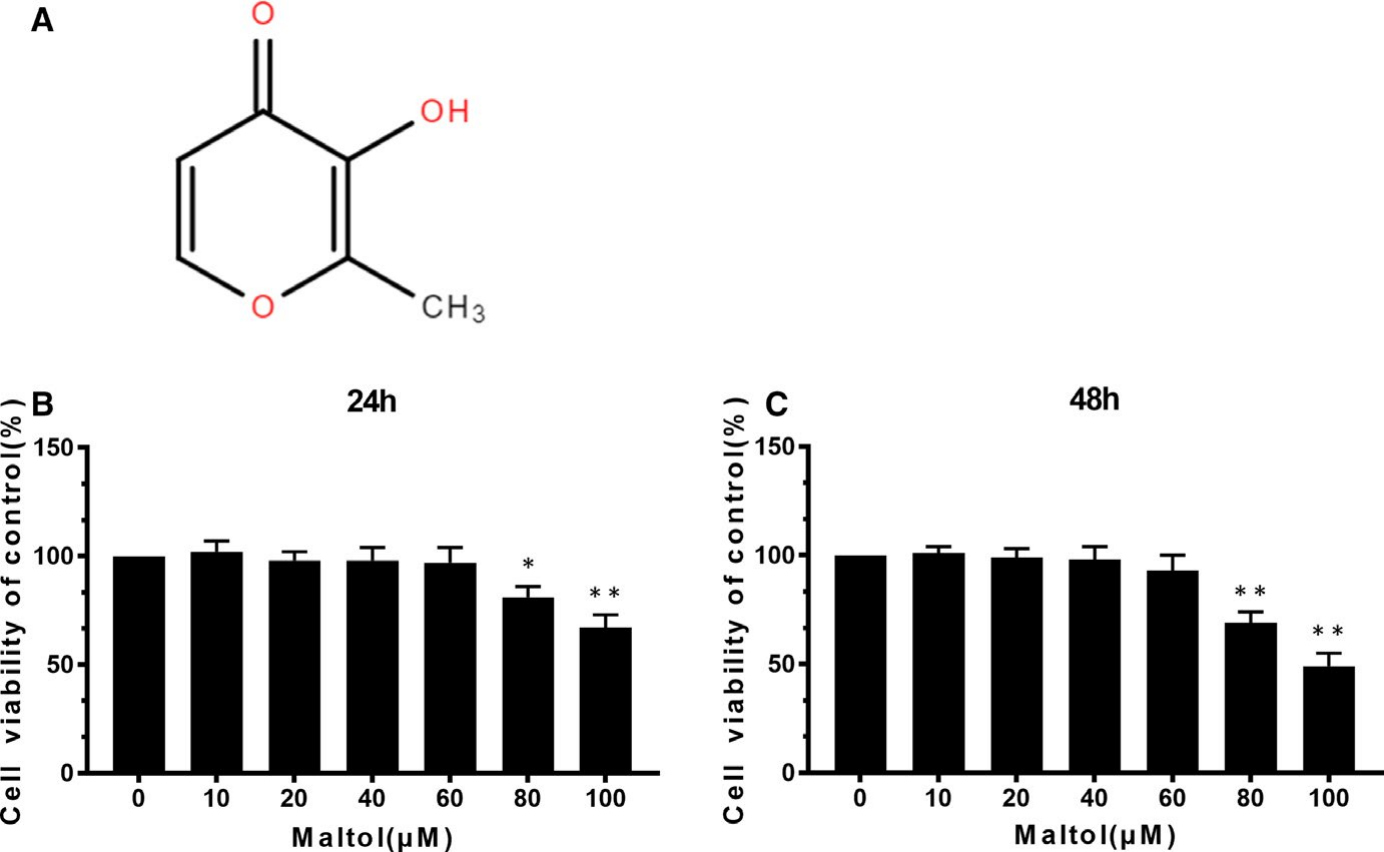
Effects of Maltol on the human chondrocyte viability. Chemical construction of Maltol (A). The cytotoxic effect of maltol (0, 10, 20, 40, 60, 80 and 100 μM) on chondrocytes for 24 and 48 h through using a CCK8 analysis (B, C). The values presented are mean values ± SD *P < .05, **P < .01 vs control group, n = 5

The chemical structure of maltol is shown in Figure 1A. Human chondrocytes were treated with various concentrations of maltol (0, 10, 20, 40, 60, 80, 100 µM) for 24 hours and 48 hours to measure the maltol cytotoxicity by CCK‐8 assay. As shown in Figure 1B,C, no cytotoxic effect of maltol were found at the dose of 0‐60 µM (*P* < .01). Whereas the cell viability was significantly decreased at 80 µM at 24 hours. Therefore, a related lower concentration of maltol (0, 20, 40 or 60 μM) was utilized for the following experiments.

### 3.2 Effect of maltol on the expression of PGE2, NO, IL‐6, TNF‐α, COX‐2 and iNOS in IL‐1β‐induced human OA chondrocytes

**Figure 2:**
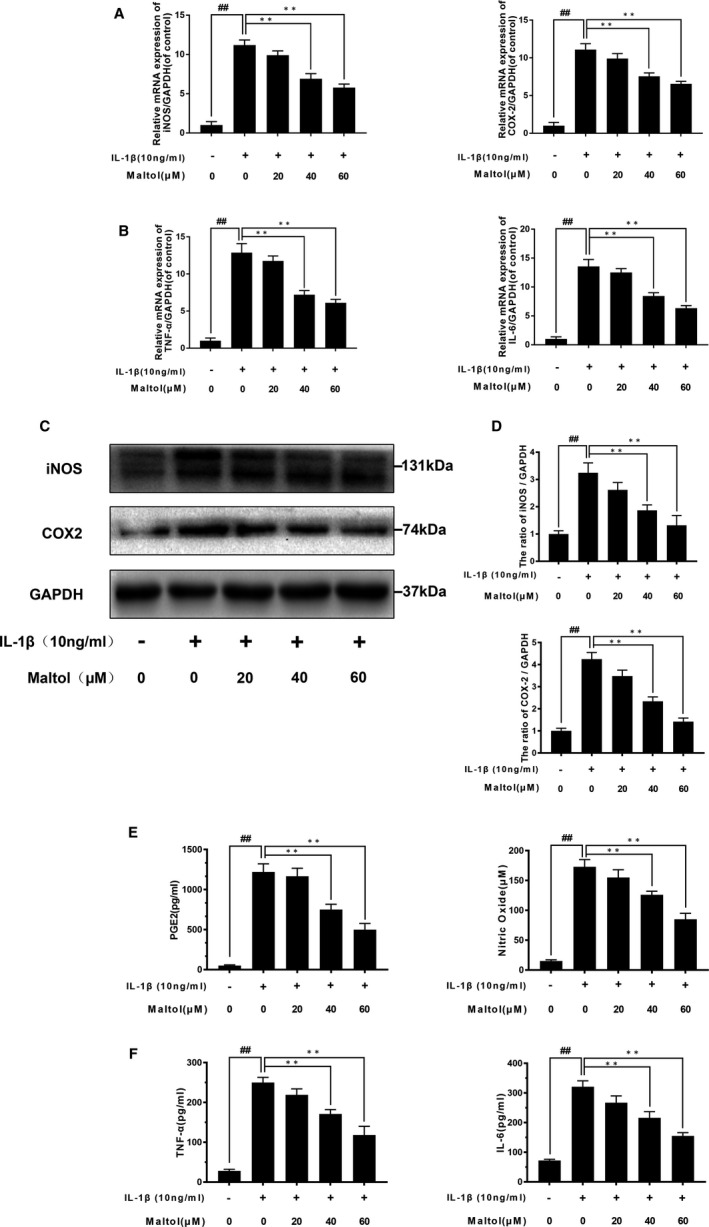
Maltol decreased the IL‐1β–induced inflammatory cytokines. The cells were pre‐treated with Maltol (20, 40 and 60 μM) before the IL‐1β irritation. The expressions of TNF‐α, IL‐6, COX‐2 and iNOS in mRNA levels were assessed by the RT‐PCR (A, B). The protein level and quantification analysis of COX‐2 and iNOS were evaluated by Western blotting (C, D). The protein level of TNF‐α, IL‐6, NO and PGE2 was assessed by ELISA analysis (E, F). Data represented are the mean values ± SD^##^ *P* < .01, vs control group; **P < .01, vs IL‐1β–alone treatment group, n = 5

We ascertained the effect of maltol on COX‐2 and iNOs induced by IL‐1β detected through qRT‐PCR and Western blotting. IL‐1β boosted the expression of COX‐2 and iNOS at protein and mRNA level (Figure 2A,C,D). Moreover, IL‐1β increased the generation of PGE2 and NO, whereas maltol could down‐regulated these inflammation mediators in a dose‐dependent manner (20, 40 and 60 μM). In addition, the results of ELISA and qRT‐PCR showed that maltol suppressed the generation of IL‐6 and TNF‐α in a dose‐dependent manner, which was increased after IL‐1β stimulation (Figure 2B,F). These results suggested that maltol could dramatically suppress the expression of IL‐1β‐induced inflammatory factors and cytokines at the protein and gene levels in a dose‐dependent manner.

### 3.3 Effect of maltol on the ECM degradation induced by IL‐1βin chondrocytes

**Figure 3:**
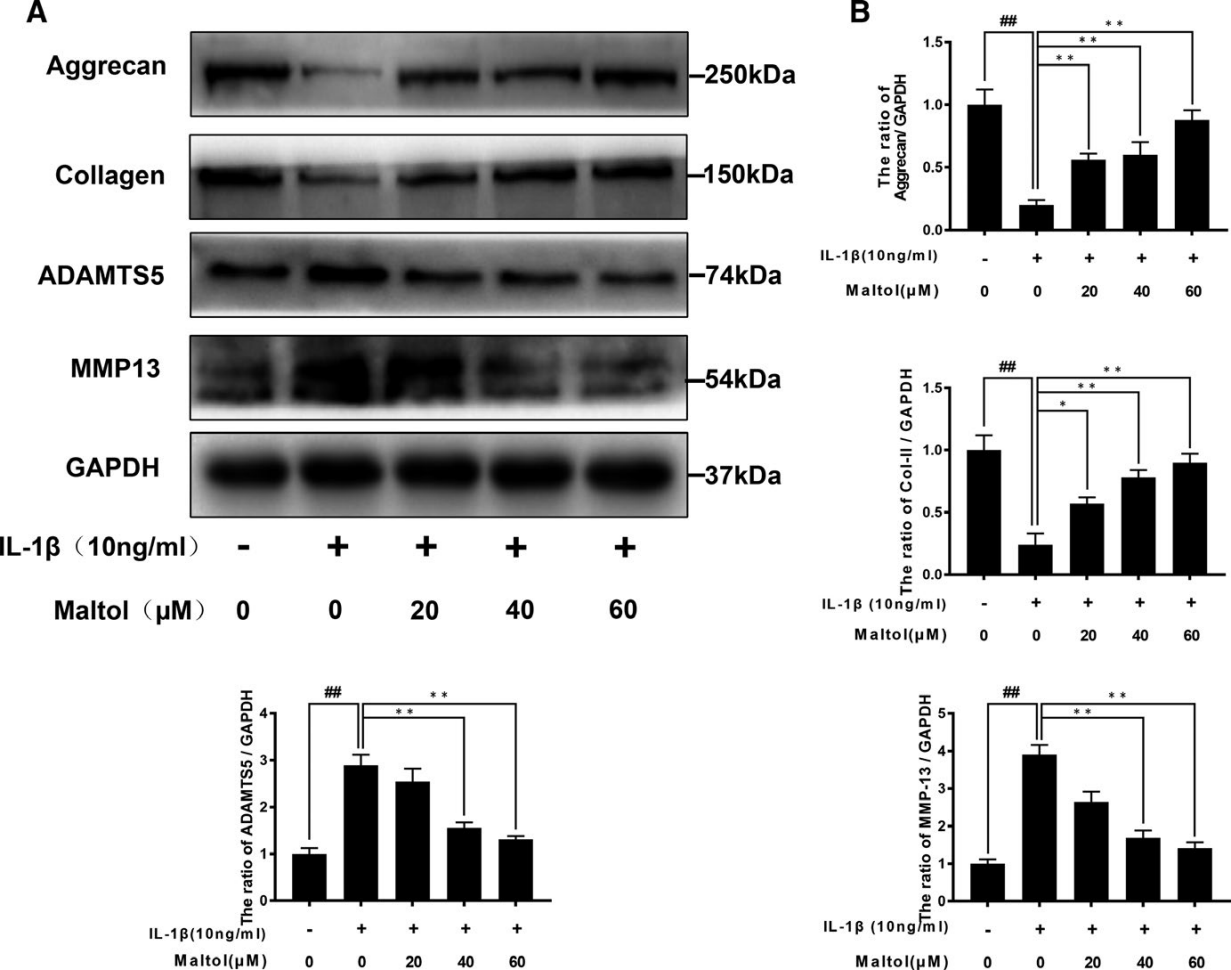
Effect of Maltol on IL‐1β‐stimulated degradation of extracellular matrix in human chondrocytes. The expressions of Collagen II, aggrecan, ADAMTS5 and MMP13 were evaluated by Western blotting (A, B). Immunofluorescence of collagen II was observed by a fluorescence microscope (OLYMPUS) (Scale bar: 50 μm) and assayed by image J (D). The data in the figures represent mean values ± SD^##^ *P* < .01, vs control group; *P < .05, **P < .01, vs IL‐1β–alone treatment group, n = 5

To determine the maltol on IL‐1β‐induced ECM degradation, we investigated the effect of maltol on aggrecan, collagen II, ADAMTS‐5 and MMP13 via using Western blotting analysis. As shown in Figure 3A,B, human chondrocytes exhibited the apparent down‐regulation of mRNA and protein expression of type II collagen and aggrecan following IL‐1β‐induced. On the contrary, IL‐1β enhanced ADAMTS‐5 and MMP‐13 production (Figure 3A,B). Nevertheless, treatment with maltol reserved the destructive effects of IL‐1β treatment in a dose‐dependent manner. Moreover, the immunofluorescence outcomes indicated that maltol attenuated the degradation of collagen II (Figure 3C,D). Therefore, these data covered that maltol could prevent ECM degradation induced by IL‐1β in human OA chondrocytes.

### 3.4 Effect of maltol on IL‐1β‐induced NF‐κB activation in chondrocytes

**Figure 4:**
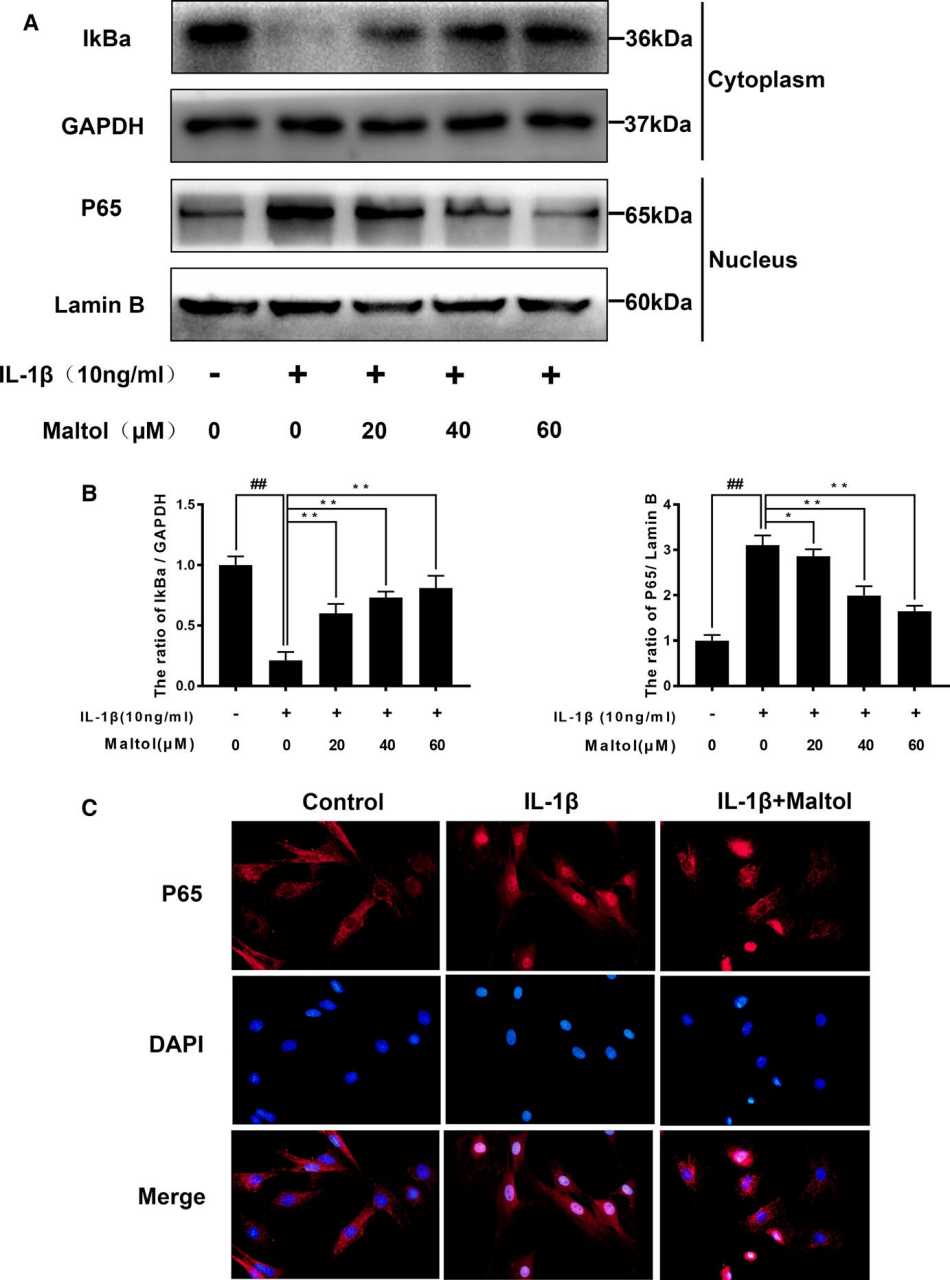
Maltol attenuated IL‐1β–induced the NF‐κB signalling pathway. The protein level and quantification analysis of p65 and IκBα in human chondrocytes were assayed by Western blotting (A‐B). Immunofluorescence of active protein of p65 was detected by a fluorescence microscope (OLYMPUS) (Scale bar: 10 μm) (C). Data represented are the mean values ± SD^##^ *P* < .01, vs control group; *P < .05, **P < .01, vs IL‐1β–alone treatment group, n = 5

To elucidate the role of maltol in anti‐inflammatory function, we utilized Western blotting to evaluate the effect of maltol on the NF‐κB signalling pathway in human chondrocytes. We, respectively, assessed the protein levels of IκBα and p65 in human chondrocytes. As shown in Figure 4A,B, IL‐1β stimulation triggered the descending of IκBα and the increase of p65, which resulted from p65 translocating from the cytoplasm into the nucleus. However, the maltol outstandingly inhibited the above action in a dose‐dependent manner (20, 40, 60 μM). Moreover, we performed immunofluorescence staining of p65 and then the image indicated that maltol mitigated the nuclear translocation of p65 induced by IL‐1β (Figure 4C). In brief, these results illustrated that maltol has an inhibitory effect on NF‐κB activation induced by IL‐1β.

### 3.5 Effect of maltol on IL‐1β‐induced PI3K and AKT phosphorylation

**Figure 5:**
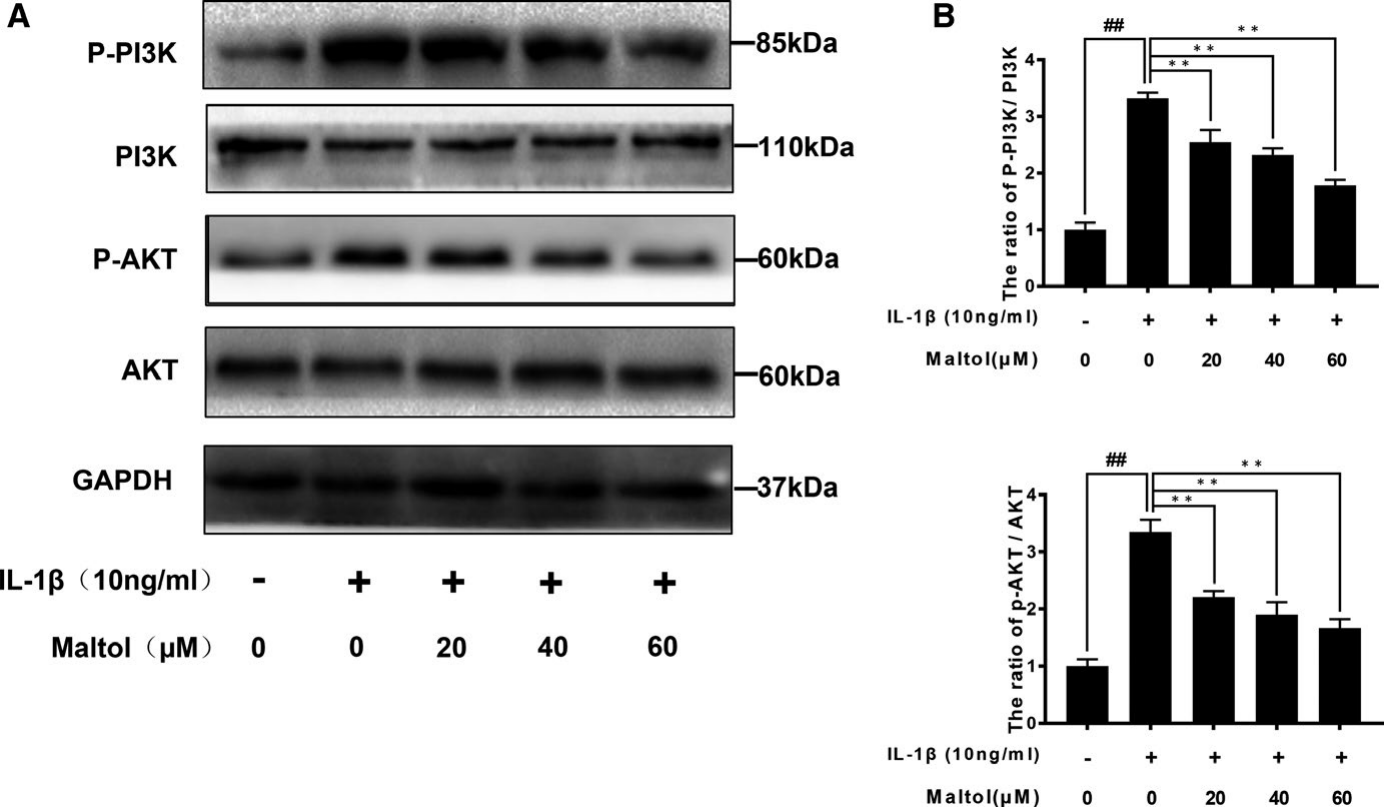
Effect of Maltol on the PI3K/Akt pathway induced by IL‐1β in human Osteoarthritis (OA) chondrocytes. The proteins expression of PI3K, P‐PI3K, Akt and P‐Akt were measured by Western blotting and quantification analysis (A, B). The data in the figures represent mean values ± SD.^##^ *P* < .01, vs control group; **P < .01, vs IL‐1β–alone treatment group, n = 5

PI3K is involved in the inflammatory induced by IL‐1β and plays a key role in the action of Akt. To determine the effects of maltol on the PI3K/Akt axis, Western blotting was adopted to evaluate PI3K and AKT phosphorylation induced by IL‐1β. As Figure 5A,B described, IL‐1β stimulation notably increased the phosphorylation of AKT and PI3K, whereas maltol treatment reversed the phosphorylation of PI3K/AKT induced by IL‐1β.

### 3.6 Maltol inhibits the degradation of cartilage in a mouse DMM model

**Figure 6:**
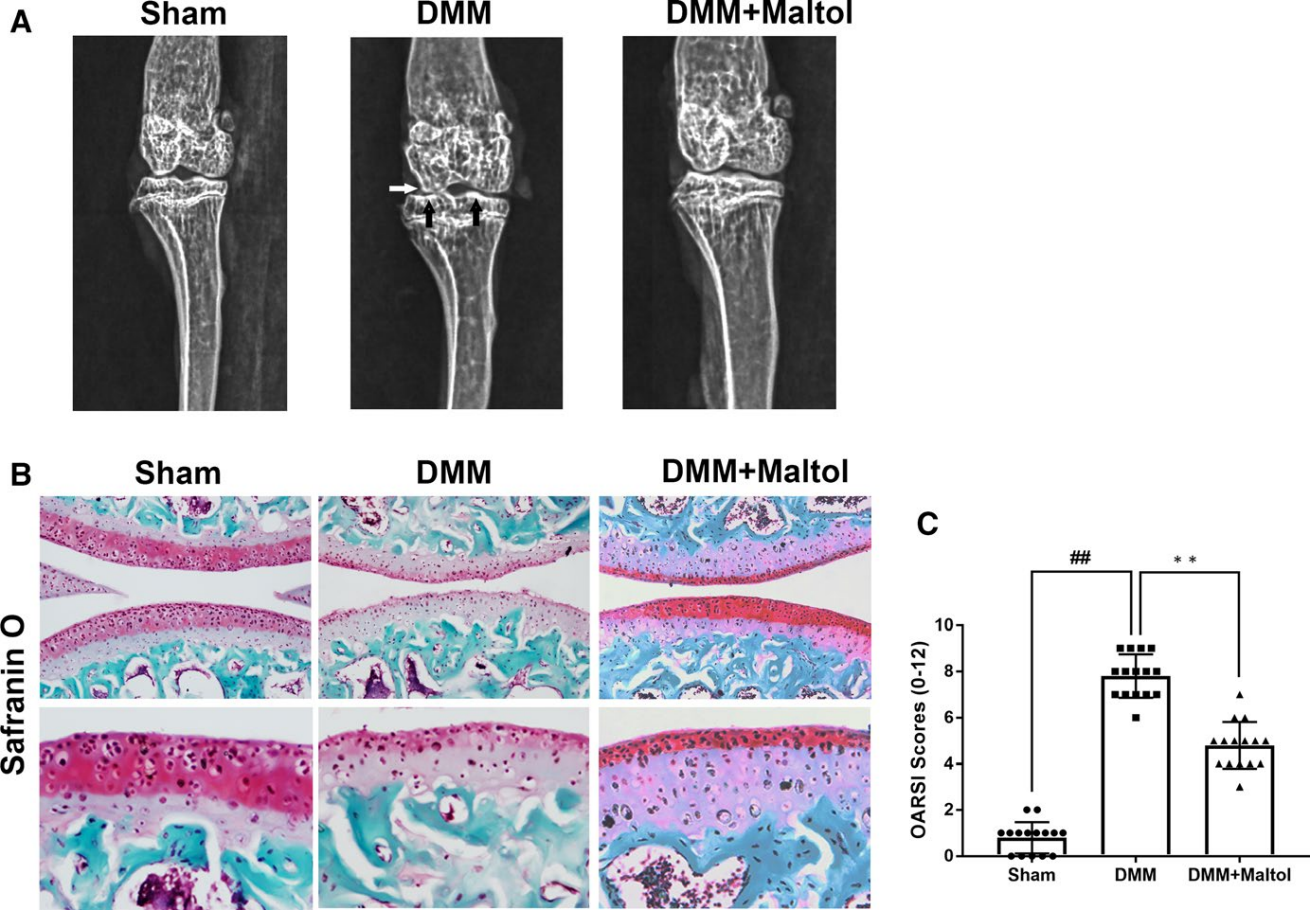
Maltol inhibits Osteoarthritis (OA) development in mouse destabilization of the medial meniscus (DMM) model in vivo. Digital X‐ray image was used to detect the narrow of joint space (white arrows) and calcification of the cartilage surface (black arrows) in OA mice (A). Typical Safranin‐O staining of the cartilage and subchondral cortical bone (scale bar: 200 μm and 50 μm) (B). Diagrams showed the cartilage OARIS scores (C). The data in the figures represent mean values ± SD.^##^ *P* < .01, vs control group; **P < .01, vs the DMM group, n = 15

We conducted a surgical operation to establish the OA mice model through destabilizing the medial meniscus. To evaluate whether maltol has the protective effects on OA progression in vivo, we used Safranin‐O staining and X‐ray to assess cartilage histological analysis of OA. Besides, the surgery increased cartilage surface density and narrowed the joint space. However, the maltol group improved the above injuries (Figure 6A). As shown in Figure 6B, maltol treatment reduced the superficial cartilage erosion and proteoglycan large loss via Safranin‐O staining. Moreover, the OARSI scores were accord with the results of S‐O staining, compared to the OA group, maltol treatment lowered the OARSI scores (Figure 6C).

### 3.7 Effect of maltol on MMP13 and Collagen II production in OA articular cartilage

**Figure 7:**
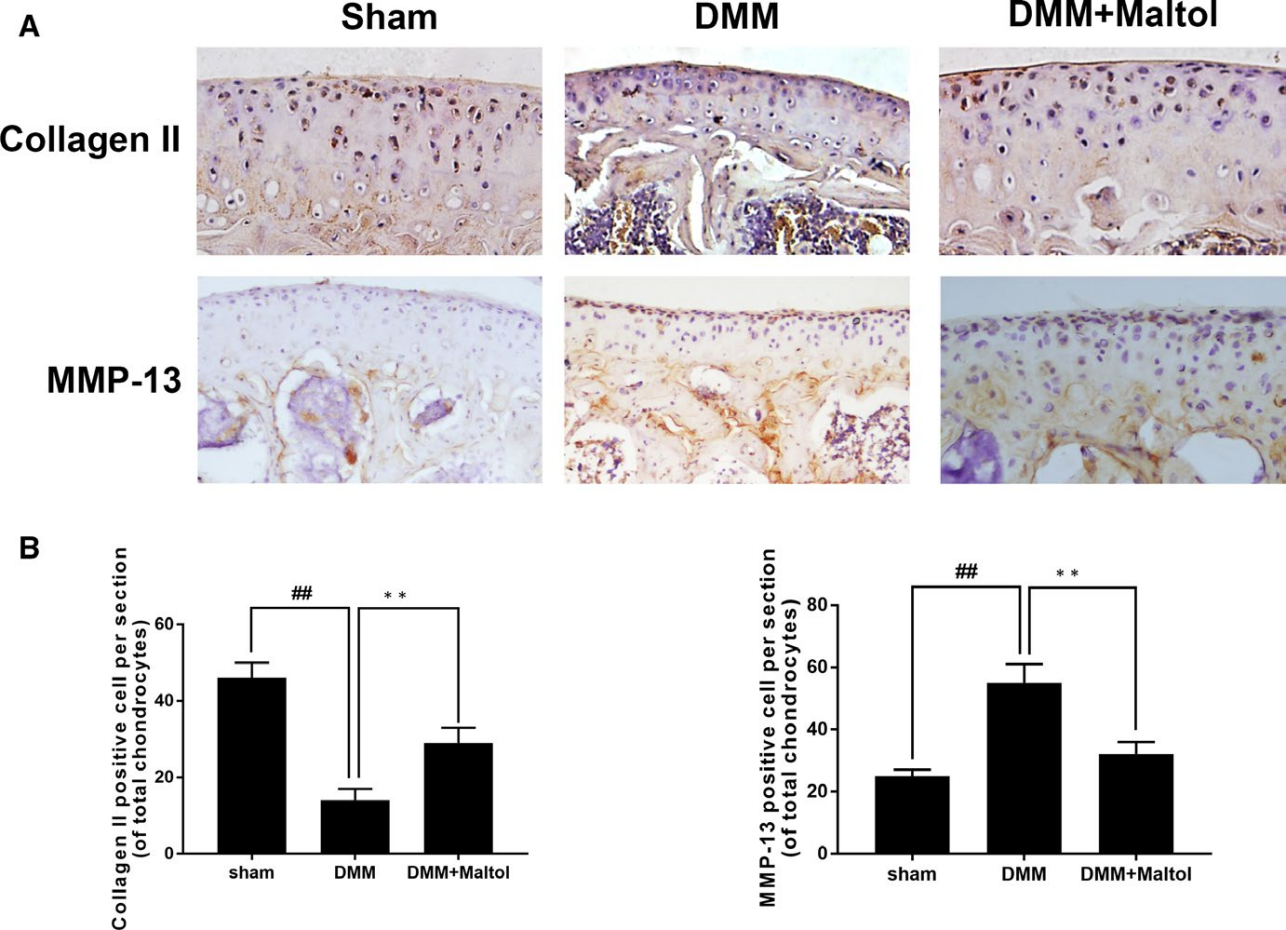
Maltol suppressed the degradation of cartilage in Osteoarthritis (OA) mice. Immunohistochemistry of MMP‐13 and Collagen II were evaluated to the effect of Maltol on the degradation of cartilage matrix in OA mice (A). Quantification of Collagen II‐positive cells and MMP‐13‐positive cells in cartilage samples (B, C). Data represented are the mean values ± SD.^##^ *P* < .01, vs control group; **P < .01, vs the destabilization of the medial meniscus (DMM) group, n = 15

To demonstrate the effects of maltol in vivo, we carried out immunohistochemical staining to detect the expression of MMP 13 and Collagen II. As illustrated in Figure 7A,B, the results of immunohistochemical staining manifested that the quantity of Collagen II in the DMM group was less than the sham group and the quantity of MMP 13 in the DMM group was more than the sham group. Fortunately, maltol treatment decayed the condition.

## 4 DISCUSSION

Various evidence has verified inflammation has a significant influence on OA.^2^, ^22^, ^23^ Hence, many anti‐inflammatory drugs had been used to retard the process of OA. Compared to non‐steroidal anti‐inflammatory drugs that only ameliorate clinical symptoms with deleterious side effects, an effective and plant‐derived compound with minor side effects have raised interest in the treatment of OA.^24^, ^25^ Maltol, a dainty food‐flavouring agent, is notable for its medicinal properties, including anti‐inflammatory, anti‐oxidative and more effects.^26^ In our study, we revealed that maltol could reduce inflammatory responses and inhibited the degradation of ECM (collagen II and aggrecan) in human chondrocytes. In addition, our study demonstrated that maltol dramatically blocks the NF‐κB pathway regulated by the PI3K/AKT signalling pathway. Moreover, maltol improved articular cartilage injury and attenuated the development of OA in OA mice models.

The NF‐κB signalling pathway, a classical pro‐inflammation pathway, has been demonstrated play a key role in the regulation of inflammatory mediators associated with OA development, such as COX‐2, iNOs and MMPs.^27^, ^28^, ^29^ The protein of P65 (a unit protein of NF‐κB) is inactive when localized in the cytoplasm combined with IκBα, an inhibitory protein.^30^ When IL‐1β stimulation, the P65 was activated and then translocated into the nucleus. Finally, the active‐P65 up‐regulates the production of inflammatory mediators.^31^, ^32^ Previous studies reported that NF‐κB p65‐specific siRNA inhibited the expression of NF‐κB p65, blocking the expression of iNOS, COX‐2 and MMP‐9 in IL‐1β‐stimulated chondrocytes.^33^, ^34^ Hence, targeted down‐regulation of NF‐κB may be deemed to cure OA effectively. Besides, the NF‐κB inhibitor could decrease the IL‐1β‐induced expression of MMP13 in human chondrocytes.^35^ As a result, in the present study, we explored whether maltol had anti‐inflammatory capability on chondrocytes with the NF‐κB signalling pathway. The data covered that maltol could dramatically suppress the phosphorylation of p65 in IL‐1β treatment in human chondrocytes. Moreover, suppressed the activity of the NF‐κB signalling pathway could down‐regulate the production of inflammatory factors, such as NO, MMPs and PGE2, to ameliorate the progression in OA. Taken together, our data, in some way, indicated that the anti‐inflammatory induced by IL‐1β of maltol on OA development and its potential mechanism was involved with the NF‐κB signalling pathway.

**Figure 8:**
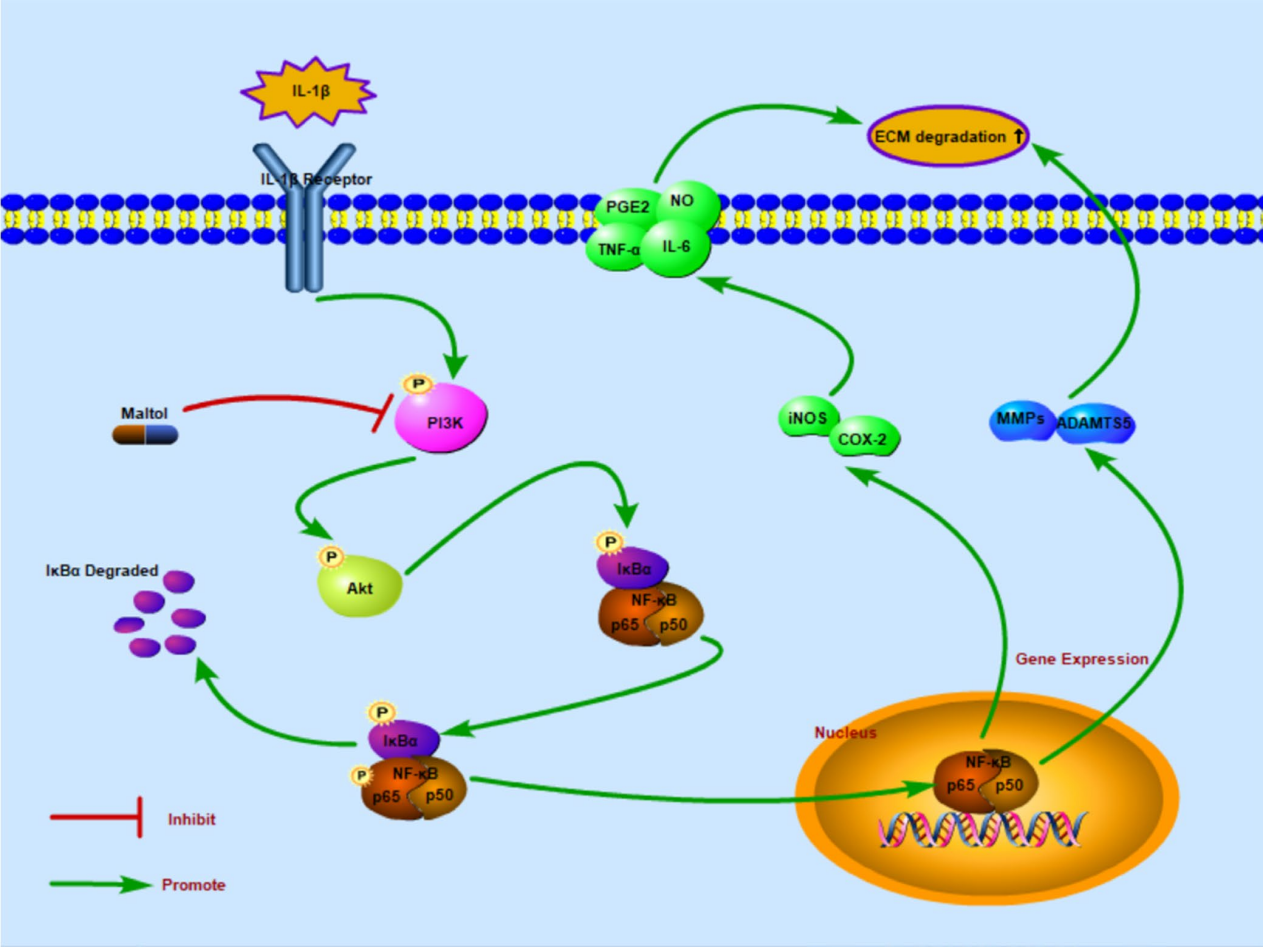
Schematic revealed that maltol suppressed the PI3K/Akt/NF‐κB pathway and potential protective effects in the progression of osteoarthritis

Accumulated evidence showed that PI3K/AKT signalling involved in the degradation of ECM and alterations of cellular in OA pathogenesis.^35^, ^36^, ^37^ Besides, previous studies demonstrated that PI3K/AKT signalling as an upstream element to regulate the activation of the NF‐κB pathway.^38^ Akt is a pivotal downstream effector of PI3K, which is an intracellular phosphatidylinositol kinase.^16^, ^39^, ^40^ The NF‐κB was suppressed via Inhibiting the PI3K/AKT pathway.^41^, ^42^ Afterwards, the NF‐κB (p65) was inhibited and subsequently abolished the expression of MMPs and COX‐2.^43^ Our previous study proved that maltol could suppress the PI3K/AKT pathway and reduced the production of inflammatory cytokine. Taken together, maltol may exert inhibitory effects in OA by preventing the IL‐1β‐induced inflammation via the PI3K/AKT/NF‐κB signalling pathway. The underlying mechanism was shown specifically in Figure 8. The potential mechanism of maltol was present.

In this study, we found that DMM groups presented a narrow joint space, severe cartilage erosion, vast proteoglycan loss and degradation of the ECM compared with the sham group. All of the above phenomena were ameliorated by treatment with maltol. Besides, the OARSI grade was lower when treated with maltol in the DMM mice model. These results and the intro findings altogether provide evidence that maltol can mitigate the progression in OA.

In conclusion, we covered that maltol could alleviate inflammatory response and ECM degradation in human chondrocytes via inhibiting the activation of the PI3K/Akt/NF‐κB pathway. Meanwhile, in surgically induced DMM mice model, treatment with maltol performed a significant role in OA progression. Taken together, our data suggest maltol exerts protective effects against OA.

## CONFLICT OF INTEREST

The authors declare that they have no conflict of interest.

## AUTHOR CONTRIBUTIONS

**Hongwei Lu:** Conceptualization (lead); Data curation (supporting); Investigation (lead); Validation (lead); Visualization (supporting); Writing‐original draft (lead). **Changchang Fu:** Conceptualization (equal); Data curation (equal); Formal analysis (equal); Resources (equal); Software (equal); Supervision (equal). **Suyan Kong:** Data curation (supporting); Resources (lead). **Xudong Wang:** Validation (lead); Visualization (supporting). **Lin Sun:** Conceptualization (supporting); Software (lead). **Zeng Lin:** Methodology (supporting); Project administration (supporting); Supervision (supporting); Writing‐original draft (lead). **Peng Luo:** Data curation (supporting); Investigation (supporting); Resources (lead). **Haidong Jin:** Formal analysis (supporting); Funding acquisition (lead); Methodology (supporting); Project administration (lead); Writing‐review & editing (lead).

## Data Availability

The data used to support the findings of the study are available from the corresponding author upon request.
